# Enhanced Pneumonia Detection in Chest x-rays using Hybrid Convolutional and Vision Transformer Networks

**DOI:** 10.2174/0115734056326685250101113959

**Published:** 2025-01-09

**Authors:** Benzorgat Mustapha, Yatong Zhou, Chunyan Shan, Zhitao Xiao

**Affiliations:** 1 School of Electronics and Information Engineering, Hebei University of Technology, Tianjin 300401, China; 2 NHC Key Laboratory of Hormones and Development, Tianjin Key Laboratory of Metabolic Diseases, Chu Hsien-I Memorial Hospital & Tianjin Institute of Endocrinology, Tianjin Medical University, Tianjin 300134, China; 3 School of Life Sciences, Tiangong University, Tianjin 300387, China

**Keywords:** Pneumonia detection, Vision transformers, Deep learning, Hyperparameter optimization, Computer-aided diagnosis, CLAHE, Convolutional neural networks, Swin transformer, Medical image processing, Chest X-ray imaging

## Abstract

**Objective:**

The objective of this research is to enhance pneumonia detection in chest X-rays by leveraging a novel hybrid deep learning model that combines Convolutional Neural Networks (CNNs) with modified Swin Transformer blocks. This study aims to significantly improve diagnostic accuracy, reduce misclassifications, and provide a robust, deployable solution for underdeveloped regions where access to conventional diagnostics and treatment is limited.

**Methods:**

The study developed a hybrid model architecture integrating CNNs with modified Swin Transformer blocks to work seamlessly within the same model. The CNN layers perform initial feature extraction, capturing local patterns within the images. At the same time, the modified Swin Transformer blocks handle long-range dependencies and global context through window-based self-attention mechanisms. Preprocessing steps included resizing images to 224x224 pixels and applying Contrast Limited Adaptive Histogram Equalization (CLAHE) to enhance image features. Data augmentation techniques, such as horizontal flipping, rotation, and zooming, were utilized to prevent overfitting and ensure model robustness. Hyperparameter optimization was conducted using Optuna, employing Bayesian optimization (Tree-structured Parzen Estimator) to fine-tune key parameters of both the CNN and Swin Transformer components, ensuring optimal model performance.

**Results:**

The proposed hybrid model was trained and validated on a dataset provided by the Guangzhou Women and Children’s Medical Center. The model achieved an overall accuracy of 98.72% and a loss of 0.064 on an unseen dataset, significantly outperforming a baseline CNN model. Detailed performance metrics indicated a precision of 0.9738 for the normal class and 1.0000 for the pneumonia class, with an overall F1-score of 0.9872. The hybrid model consistently outperformed the CNN model across all performance metrics, demonstrating higher accuracy, precision, recall, and F1-score. Confusion matrices revealed high sensitivity and specificity with minimal misclassifications.

**Conclusion:**

The proposed hybrid CNN-ViT model, which integrates modified Swin Transformer blocks within the CNN architecture, provides a significant advancement in pneumonia detection by effectively capturing both local and global features within chest X-ray images. The modifications to the Swin Transformer blocks enable them to work seamlessly with the CNN layers, enhancing the model’s ability to understand complex visual patterns and dependencies. This results in superior classification performance. The lightweight design of the model eliminates the need for extensive hardware, facilitating easy deployment in resource-constrained settings. This innovative approach not only improves pneumonia diagnosis but also has the potential to enhance patient outcomes and support healthcare providers in underdeveloped regions. Future research will focus on further refining the model architecture, incorporating more advanced image processing techniques, and exploring explainable AI methods to provide deeper insights into the model's decision-making process.

## 1 INTRODUCTION

Pneumonia, a respiratory infection caused by either viral or bacterial pathogens, affects individuals across all age groups and manifests in varying degrees of severity. It remains the primary cause of mortality in the global pediatric population and poses a significant threat to individuals aged 65 and above, as well as those with preexisting medical conditions. In humans, the lungs contain tiny sacs called alveoli, which fill with air during healthy breathing. When pneumonia occurs, these alveoli become filled with pus and fluid, causing discomfort and difficulty in breathing and restricting oxygen intake. Infections are commonly transmitted through direct contact with infected individuals. Statistically, pneumonia impacts approximately 1,400 children per 100,000, contributing to 14% of fatalities among children below five years of age in 2019, resulting in the deaths of 740,180 children [1]. In 2017, pneumonia caused over 808,000 deaths among children under five, representing 15% of all fatalities in this age group [2]. The reduction in these figures can be attributed to improved detection techniques and the development of health technologies such as vaccines.

However, the number of pneumonia-related deaths among older adults has increased over time despite a reduction in environmental pollution [3]. Timely identification of pneumonia is crucial for improving treatment outcomes and reducing mortality rates. Diagnosis typically depends on an individual's medical history and the severity of the condition. The primary diagnostic tool for pneumonia is chest X-rays, where areas of infection are identified by white patches in the pulmonary region. Experience is essential for identifying affected areas in radiographic images and determining the need for further radiology examination [4]. While chest X-rays are common, they present challenges, particularly in patients aged 65 and older, where obtaining good-quality images can be difficult [5]. Computed tomography (CT) scans offer enhanced visualization of lung structures and other critical anatomical regions, including the airways. In specific scenarios, CT scans may serve as a preferable substitute for chest X-rays. However, both procedures entail exposure to ionizing radiation, which carries a minimal but inherent risk of cancer [6, 7]. Detecting pneumonia using chest X-rays or CT scans is challenging even for professional radiologists and requires significant experience. The interpretation of pneumonia in x-ray and CT scan images often presents challenges due to inherent ambiguity, potentially leading to misdiagnosis by attributing findings to other diseases or common illnesses. Despite the expertise of trained professionals, the human element introduces the potential for errors in the analysis and interpretation of imaging results. This emphasizes the need for a computer-aided diagnostic model that is both accurate and immediate. Pneumonia detection involves classifying data into two categories: pneumonia or normal, framing it as a classification problem.

CNN approaches outperform traditional methods by automatically extracting intricate features from input images, eliminating the need for manual feature engineering. This capability has made CNNs highly popular for image classification across various medical domains, including oncology, dermatology, and radiology [8-10]. For instance, in oncology, CNNs have been effectively utilized for tumor detection and classification in mammograms and histopathological images, demonstrating superior accuracy compared to conventional image processing techniques [11]. Additionally, in dermatology, CNN-based models have achieved dermatologist-level performance in classifying skin lesions, aiding in early diagnosis and treatment planning [12]. These advancements underscore the versatility and effectiveness of CNNs in enhancing diagnostic precision across multiple fields of medicine.

The CNN model examines connections between neighboring pixels within a specific receptive field defined by the filter size. Despite its effectiveness in capturing local spatial hierarchies, it struggles with recognizing relationships between distant pixels, which hinders its ability to understand the broader context and more complex patterns within an image. To overcome this limitation, recent advancements have integrated attention mechanisms, allowing the model to dynamically weigh the importance of pixels, regardless of their distance from each other. This enhancement helps capture both local and global dependencies more effectively.

Researchers have extensively explored various image enhancement techniques to boost the classification accuracy of deep learning models in medical imaging. Techniques such as adaptive histogram equalization (AHE) and its variant, CLAHE, have been widely adopted to enhance image contrast and highlight critical features [13]. Additionally, normalization methods like z-score normalization and min-max scaling are commonly employed to standardize image intensities, thereby reducing variability and improving model convergence [14, 15]. Denoising techniques, including Gaussian filtering, median filtering, and more advanced methods like Non-Local Means (NLM) and Wavelet-based denoising, have been utilized to remove unwanted noise while preserving essential anatomical structures [16, 17]. Furthermore, advanced normalization methods such as batch normalization and instance normalization have been integrated into deep learning architectures to stabilize and accelerate training processes [18]. These enhancement and normalization strategies facilitate better feature extraction by neural networks, leading to improved classification performance. Studies have reported that incorporating these techniques results in significant improvements in diagnostic accuracy, sensitivity, and specificity compared to using raw images alone [9, 19].

Moreover, attention mechanisms have been applied in medical imaging, particularly in identifying lung diseases from chest x-ray images. Attention mechanisms, recognized for their ability to balance and accurately extract relevant features, have shown promising results in enhancing diagnostic accuracy and reliability in medical imaging [20, 21]. By dynamically focusing on pertinent regions of an image, attention modules improve the model's sensitivity to critical pathological indicators, thereby facilitating more precise diagnoses [22]. For example, attention-based models have been successfully applied in tasks such as tumor segmentation in MRI scans and lesion detection in retinal images, demonstrating superior performance compared to traditional CNNs [23, 24]. These advancements highlight the potential of attention mechanisms to refine feature extraction processes and contribute to more reliable computer-aided diagnostic systems.

Despite these advancements, there remains a significant gap in research on recent Multi-Head Self-Attention (MSA) methods. These methods, which involve multiple attention heads focusing on different parts of the image simultaneously, have shown greater potential in computer vision tasks. As these methods continue to evolve, they are expected to offer even greater improvements in performance and capability, paving the way for more advanced and accurate models across various applications.

Considering these disadvantages, this study presents a comprehensive framework for pneumonia identification, capable of handling binary classification tasks and implements one stage of the swin transformer. Specifically, it focuses on patch extraction, embedding, merging, and expanding, which are part of the swin transformer's hierarchical processing, combined with window-based self-attention, shift operations, and MLP operations. Implementing a stage of the swin transformer allows for enhanced feature representation, improved information integration, and effective hierarchical processing, leading to a better understanding of complex visual patterns.

This study emphasizes the significant impact of pneumonia in underserved regions, where the limited availability of accurate and accessible diagnostic tools hinders timely treatment. Our innovative technique aims to address this issue by offering a lightweight, deployable solution that does not rely on extensive hardware. The key contributions of this research are as follows:

(1) The integration of swin transformer blocks into the CNN model architecture is a significant advancement, leveraging the strengths of transformers in capturing global context and dependencies within image data, enhancing the model's ability to understand complex structures.

(2) The introduction of patch extraction and embedding layers represents a novel approach to image processing. By breaking images into smaller patches and processing them sequentially, the model effectively handles large images and those with intricate details.

(3) Utilizing optuna for hyperparameter tuning is a major contribution, as it efficiently explores the hyperparameter space to find the optimal configuration. The use of bayesian optimization (TPE sampler) further improves the efficiency and effectiveness of this search process.

(4) The model architecture's robustness is enhanced by including various hyperparameters related to both convolutional and transformer layers. This comprehensive search space allows for fine-tuning the feature extraction capabilities of convolutional layers and the attention mechanisms of transformer layers, ensuring flexibility and robustness.

(5) Implementing k-fold cross-validation contributes to a robust evaluation of the model by training and validating it on different subsets of data. This method leads to more reliable performance metrics. Achieving an average accuracy of 98.016% across folds highlights the model's strong performance and generalization capability.

The paper is structured as follows: Section 2 provides a review of previous attempts to diagnose pneumonia using deep learning. Section 3 entails a thorough examination of the research methodology and the employed dataset. Section 4 delves into the experimental setup and the outcomes of the performance evaluation. The concluding segment, Section 5, summarizes the key findings and serves as the conclusion of the paper.

## 2 RELATED WORK

The classification of pneumonia is crucial for assessing the disease and determining treatment options based on its different types. Pneumonia is typically diagnosed using various medical imaging techniques, with chest X-rays being commonly used due to their efficiency and cost-effectiveness. On the other hand, deep learning (DL) has emerged as a leading technology in research, known for its capacity to process large amounts of data effectively. DL is a machine learning method that has consistently shown impressive results, especially in tasks related to categorization and detection. Fig. (**1**) displays a tree diagram of relevant studies on pneumonia classification. Table **1** showcases a comparative analysis of deep learning techniques.

The use of deep learning techniques, particularly Convolutional Neural Networks (CNNs), Transfer Learning (TL), and Vision Transformers (ViTs), has been extensively explored for diagnosing pneumonia from medical images. Each approach offers unique advantages in terms of accuracy, generalizability, and computational complexity, as detailed below.

Stephen *et al*. [25] developed and trained a CNN model specifically tailored to their dataset from inception. The model attained a peak training accuracy of 95.31% and a validation accuracy of 93.73%. Furthermore, the preprocessing steps encompassed the implementation of various data augmentation techniques. Yue *et al*. [26] employed CT scan images to analyze six distinct features for diagnosing pneumonia. The model obtained an impressive AUC score of 97%. Nevertheless, it is crucial to highlight that the assessment was executed on restricted datasets, indicating potential limitations in generalizability. DL models rooted in CNN architectures typically outperform conventional machine learning models.

In their network backbone, Liang *et al*. [27] integrated residual blocks and dilated convolution layers into their network backbone, leading to a recall rate of 96.7% and an F1-Score of 92.7%. Yadav *et al*. [28] presented a CNN model that attained an accuracy of 84.18%, recall of 78.33%, precision of 94.05%, and F1-score of 85.66% in the realm of classification performance.

A specialized CNN architecture was proposed by Harsh *et al*. [29] for the purpose of diagnosing pneumonia, which was subsequently expanded as ensembles featuring various convolutional filter sizes. The training of the CNN model involved 3875 pneumonia images and 1341 images categorized as normal. The outcome yielded a recall rate of 99.23%, albeit with noticeable inaccuracies and signs of overfitting. Ebru *et al*. [30] introduced a deep learning framework tailored for the identification of pneumonia in lung scans, resulting in an overall accuracy of 88.62% and a recall rate of 97.43%.

In their study, Urey *et al*. [31] executed preprocessing techniques on chest x-ray images through distinct methodologies, followed by the application of three different network models. In the feature extraction part, they utilized the CNN model to derive feature maps from the preprocessed images, employing techniques such as image contrast enhancement and unpacking. Subsequently, the chest images were categorized into normal, bacterial pneumonia, and viral pneumonia, achieving an overall classification accuracy rate of 79%. Zhang *et al*. [32] introduced a confidence-aware module designed for effective anomaly detection in chest X-ray images. This model excelled in locating irregularities, boasting an impressive AUC score of 83.61%.

Transfer Learning has been used to overcome the limitations of training deep learning models on small medical datasets. The study conducted by Chouhan *et al*. [33] employed the Guangzhou Women's and Children's Medical Center and implemented a transfer learning algorithm, resulting in an accuracy rate of 96.4%. Rajpurkar *et al*. [34] utilized the DenseNet-121 CNN model to scrutinize the ChestX-ray14 dataset, comprising 112,150 frontal chest x-ray images. The model attained a score of 76.8% on the f1 test.

On the other hand, Jain *et al*. [35] achieved an accuracy of 95.62%, recall of 95%, and precision of 96% for pneumonia detection from chest x-ray images through the utilization of CNN and transfer learning. Asnaoui, Chawki, and Idri utilized E3CC and VGG16 + CapsNet in their study [36]. E3CC obtained an accuracy rate of 81.54%, whereas VGG16 + CapsNet reached an accuracy rate of 88.30%.

Meanwhile, in a separate study, Mittal *et al*. [37] realized an accuracy of 85.26%, recall of 94%, and F1-score of 89%, employing the Simple CapsNet model. Summerly, Ayan, *et al*. [38] enhanced the performance of two classical CNN architectures (VGG16 and Xception) *via* transfer learning for the classification of pneumonia disease, resulting in an accuracy rate of 87%. Kundu *et al*. [39] utilized an ensemble model encompassing three distinct transfer learning architectures, namely GoogLeNet, ResNet-18, and DenseNet-121. The final iteration of the proposed model achieved an accuracy of 86.85%. Nonetheless, the integration of three transfer learning models into an ensemble technique leads to a noticeable escalation in the computational requirements.

O'Quinn, *et al*. endeavored to detect the presence of Pneumonia utilizing the DICOM format, as delineated in [40]. By employing the transfer learning technique, AlexNet achieved recognition accuracy of 76%. Talo *et al*. [41] employed the ResNet152 model to identify pneumonia disease *via* the transfer learning technique. They successfully recognized 97.4% of the dataset without necessitating any preprocessing or feature extraction.

However, Varshni *et al*. utilized DenseNet as a feature extractor and employed an SVM classifier on the inputs processed by DenseNet [42]. The dataset employed was ChestXray14. Before addressing the challenge with DenseNet, multiple transfer learning modules were experimented with. The model yielded an AUC value of 80.02%.

Regrettably, the diagnosis of pneumonia through X-rays remains a substantial challenge, even for proficient and knowledgeable medical practitioners, due to the fact that x-ray images offer similar area information for other ailments, such as lung cancer. Consequently, the process of diagnosing pneumonia utilizing traditional methods is both laborious and resource-intensive, posing difficulties in establishing a standardized approach for determining the presence of pneumonia in a patient. Numerous researchers have devoted their endeavors to enhancing the efficacy of CNN and have effectively demonstrated significant progressions over time.

In contrast, the CNN model scrutinizes solely the interplay between neighboring pixels within the delimited receptive region determined by the filter dimensions. Consequently, establishing relationships with distant pixels is notably challenging. Recent endeavors have been undertaken to integrate attention mechanisms as a solution to tackle this obstacle. Attention, as a methodology, is employed for the purpose of detecting and concentrating on the most pertinent and insightful segment of the information.

The attention mechanism [43-45] is currently considered a state-of-the-art advancement in tasks related to computer vision. It surpasses conventional CNN models by selectively highlighting important features while ignoring redundant ones. In contrast to CNNs, which process the entire input image and may, therefore, focus on both essential and non-essential information, the attention mechanism helps mitigate false-negative outcomes. By utilizing the attention mechanism, not only is the interpretation enhanced, but the classifier's performance is also optimized without adding extra computational burden to the model.

Guo *et al*. [46] proposed the adoption of an abnormal-aware attention mechanism to transform low-level features of an input image into high-level features, considering the importance of each feature. This led to the development of a framework that dynamically assigns a score of attentiveness to each connected dense layer. Additionally, they implemented a unique angular contrastive loss function to reduce the loss within the same class and increase the loss between different classes. Their technique achieved an accuracy rate of 89.4% in WCE images, thereby incorporating spatial and channel attention into deep learning models for the purpose of pneumonia detection.

Zhang *et al*. [45] introduced a new Shuffle Attention mechanism to address the issue of computational complexity. This mechanism applies both channel and spatial attention to input features, resulting in enhanced classification performance on ImageNet-1k with an accuracy improvement of over 1.34%. This strategy exhibits superior performance in the medical domain due to its capacity to capture intricate details of minor lesions.

Transformers [20, 47] are intricate neural network architectures that make use of attention mechanisms. Initially designed for tasks associated with natural language processing (NLP), they have now become a source of inspiration for researchers in the computer vision field after achieving outstanding performance in NLP tasks. Researchers are interested in exploring the potential of transformers in visual challenges due to their capability to capture long-range dependencies within an image [48]. The VIT has effectively implemented the transformer model in image processing, demonstrating favorable performance in image classification assessments when compared to cutting-edge CNN [49].

Sukhendra *et al*. [50] proposed a novel framework for detecting pneumonia using the VIT architecture on chest X-rays. Their approach leverages the VIT model to capture global context and spatial relationships within the X-ray images, achieving a high accuracy of 97.61%, along with a sensitivity of 95% and specificity of 98%. On the other hand, Usman *et al*. [51] demonstrated that the Vision Transformer applied on the pediatric pneumonia dataset, achieved superior performance across multiple evaluation metrics, with a precision of 0.89, recall of 0.84, F1-score of 0.86, AUC of 0.87, and accuracy of 0.87, outperforming other CNN-based models such as ResNet-50, Inception-V3, and VGG-16.

To address the challenge of overfitting due to a limited number of training samples, our hybrid architecture is structured into two distinct phases. First, we employed the proposed CNN layers to extract common deep features. In the second phase, these extracted features were further analyzed and used for predicting pneumonia in chest x-ray images *via* a swin transformer block and a neural network. Despite the constraint of limited training samples, our model consistently demonstrated robust generalization across various datasets, as evidenced by its performance in different cross-validation scenarios and tests on unseen data.

## 3 METHODOLOGY

We have developed a method to determine the prognosis of pneumonia using image data collection. Our approach involves preprocessing the data and employing a hybrid CNN architecture. We refined this method by adjusting hyperparameters across various layers, including convolutional layers, global average pooling, swin transformer block layers, and dense layers. Using a dataset of pneumonia x-ray images, we applied our algorithm to detect pneumonia. The block diagram and design of our proposed framework are illustrated in Fig. (**2**). The suggested methodology progresses through a series of steps in the following manner:

(1) Step 1: In the initial step, the pneumonia dataset is utilized, consisting of two categories: normal and pneumonia.

(2) Step 2: During the image preprocessing stage, all images are resized to 224x224 pixels. To enhance image features, CLAHE is applied. Additionally, the image data are standardized for consistency.

(3) Step 3: The dataset is then split into training (80%), validation (12.7%), and test (7.3%) sets, with the test set consisting of unseen data, to ensure robust evaluation and generalization of the model.

(4) Step 4: To prevent overfitting and address dataset imbalance, an image data generator is used to apply various transformations, including zoom, rotation, and horizontal flip.

(5) Step 5: The model training process involves training the hybrid CNN model, which has a swin transformer block, which performs advanced feature extraction using patch extraction, embedding, merging, expanding, window-based self-attention, shift operations, and MLP operations. The output is subsequently passed through custom classification layers for prediction.

(6) Step 6: The model is trained using the training data and validated with the validation data. Callbacks such as learning rate reduction, early stopping, and model checkpointing are employed to optimize training.

(7) Step 7: Finally, the model's performance is evaluated using various metrics, including accuracy, precision, recall, F1-score, and the confusion matrix.

### 3.1 Data Collection

The dataset employed in this study was obtained from Kaggle as an openly accessible dataset, graciously provided by the Guangzhou women and children's medical center in Guangzhou. Additional normal cases were introduced to create a balanced dataset. Prior to commencing the analysis, any unreadable or substandard images were eliminated. Following this, two expert medical practitioners evaluated the diagnoses of the remaining images. To promote equilibrium in the machine learning task, modifications were made to the dataset by adjusting the number of instances designated for training and validation purposes [52].

To facilitate a more even-handed machine learning undertaking, modifications were made to the dataset by redistributing the quantity of x-ray images designated for training and validation purposes. The dataset consists of a total of 8,525 images in JPEG format. These images are sorted into three categories: training, validation, and testing. The training category consists of 6,820 images, the validation category comprises 1,080 images, and the testing category is made up of 625 images. The distribution of the dataset is depicted in Fig. (**3**).

### 3.2 Data Preprocessing

The dataset was carefully prepared for preprocessing by extracting both images and label information. This preparation included organizing folders using the labels as identifiers. The image preprocessing phase involved resizing the images to 224x224 pixels and applying CLAHE. CLAHE is a technique that enhances local contrast to improve detail visibility across different regions of an image.

After conducting numerous experiments with various filters, such as histogram equalization with gaussian blur, bilateral filter, and Laplacian filter, it was determined that CLAHE was the optimal choice for enhancing image contrast. The dataset was then divided into training, testing, and validation subsets with proportions of 80%, 12.7%, and 7.3%, respectively, to accommodate the Hybrid-CNN model.

The preprocessed images exhibited sharper, brighter, and more distinct details compared to the originals, making them suitable for model input. The CLAHE parameters were chosen with a clip limit of 0.02 and a tile grid size of 8x8 based on the results of hyperparameter optimization conducted using the bayesian optimizer. The outcome of the filter application is illustrated in Fig. (**4**).

### 3.3 Data Augmentation

Data augmentation is a widely adopted technique to increase the size and diversity of a dataset, thereby enhancing the performance and generalization capabilities of deep learning models [53, 54]. In our proposed approach, we applied various image processing methods to generate augmented images from the originals. This augmentation involves operations like horizontal flipping, rotating, and zooming. To streamline the training, the input images are rescaled to a range of 0 to 1.

Starting with the preprocessed images, we implemented the following augmentation techniques: the horizontal flip technique randomly mirrors the input image horizontally. It is applied to images with dimensions of 224x224, where 224 represents the image's height and width. The random rotation technique randomly rotates the input image between -15 and +15 degrees. The random zoom technique applies a random zoom effect to the input image, with a maximum zoom level of 20%.

Our augmentation process was designed and adjusted to ensure that the augmented dataset maintains diversity and reflects the characteristics of the original dataset. The proposed model was trained using this augmented dataset. Table **2** details the corresponding augmentation parameters.

### 3.4 Model Architecture

The architecture of our hybrid model integrates the strengths of CNNs and transformer-based self-attention mechanisms to achieve superior performance in image classification tasks. The model begins with a robust CNN backbone that efficiently extracts low-level features from the input images. The input layer accepts images of shape (224, 224, 1). The backbone comprises four convolutional blocks, each followed by ReLU activation and max-pooling layers to downsample the spatial dimensions while expanding the feature depth.

The first block employs a convolutional layer with 96 filters of size (5, 5), followed by max-pooling with a size of (2, 2). The second block includes a convolutional layer with 192 filters of size (3, 3) and similar max-pooling. The third block consists of a convolutional layer with 336 filters of size (3, 3) and max-pooling, and the fourth block has a convolutional layer with 480 filters of size (5, 5) and max-pooling. This hierarchical extraction of features ensures that the network captures both fine-grained and coarse patterns from the images. Padding is applied to maintain spatial dimensions for subsequent operations.

Following the CNN backbone, the feature maps undergo patch extraction using the patch extraction layer, which divides the feature maps into non-overlapping patches of size (2, 2). These patches are then embedded into a higher-dimensional space using the patch embedding layer, which combines linear projection and positional embedding to retain spatial information.

The core of the model comprises four swin transformer blocks that perform self-attention within local windows. Each block executes MSA on (2, 2) windows, with cyclic shifts to introduce cross-window connections. This attention mechanism allows the model to capture long-range dependencies effectively. Within each block, an MLP layer with two dense layers and gaussian error linear units (GELU) activation is applied, along with layer normalization before the attention and MLP layers. Drop path regularization is incorporated to mitigate overfitting and enhance training stability by introducing stochastic depth.

After processing through the Swin transformer blocks, the patches are merged using the patch merging layer, which reduces the spatial dimensions while increasing the feature depth. This step aggregates the local features into a more compact representation.

To produce the final classification, global average pooling (1D) is applied to the merged patches, converting them into a fixed-size feature vector. This vector is then passed through a series of fully connected layers. The first dense layer contains 384 units with ReLU activation, followed by a dropout layer to prevent overfitting. The subsequent dense layers have 160 and 64 units, each followed by ReLU activation and dropout layers. The output layer, a single-unit dense layer with a sigmoid activation function, performs binary classification.

The model is compiled using the Adam optimizer, binary cross-entropy loss function, and accuracy metric. This hybrid architecture leverages the local pattern recognition capabilities of CNNs and the global contextual understanding of self-attention mechanisms, making it highly effective for image classification tasks. The architecture of the hybrid model is illustrated in Fig. (**5**).

### 3.5 Hybrid Swin-T block

The swin transformer block is formulated through the substitution of the conventional MSA component, commonly present in a Transformer block, with a component that depends on shifted windows [55]. Incorporating a shifted window-based MSA module, the structure of a swin transformer block consists of a 2-layer MLP with GELU nonlinearity nested in between. Preceding each MSA module and MLP, a LayerNorm (LN) layer is incorporated, and a residual connection is introduced subsequent to each module, as illustrated in Fig. (**6**).

#### 3.5.1 Self-attention in Non-overlapped Windows

Self-attention in non-overlapping windows is utilized to enhance the modeling process in the context of the swin transformer. The calculation of self-attention is performed within specific local windows that are designed to partition the image evenly, devoid of any overlapping sections. It is assumed that each window consists of *M* x *M* patches. The computational complexity of a universal MSA module and a window-based approach can be approximated for an image containing *h* x *w* patches:


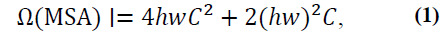



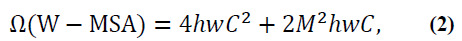


#### 3.5.2 Shifted Window Self-attention

The self-attention module relying on windows lacks interconnections between these windows, thereby impeding its efficacy in modeling. To overcome this constraint while upholding the efficient processing of non-overlapping windows, the swin transformer adopts a window partitioning shifting approach. This strategy entails switching between two partitioning configurations in consecutive swin transformer blocks to incorporate inter-window links. By applying the shifted-window partitioning technique, the calculations for successive swin transformer blocks proceed as follows:


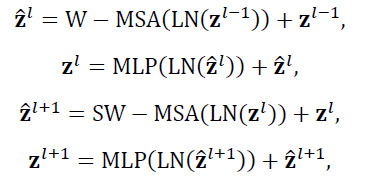


#### 3.5.3 Multilayer Perceptron

The MLP is a neural network model characterized by its feed-forward architecture, which includes dense and dropout layers [20]. Within the context of this investigation, the MLP is specifically designed with two non-linear layers that utilize GELU. The arrangement of the MLP blocks remains uniform, utilizing stacked layers with comparable configurations. For example, let 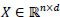 symbolize token attributes, where *n* represents the sequence length and *d* signifies the dimension. Each block is defined mathematically as:


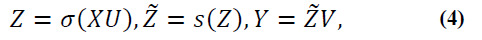



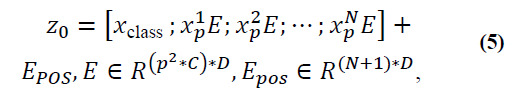



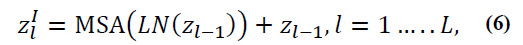



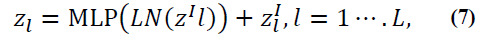



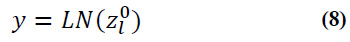


W-MSA and SW-MSA refer to window-based multi-head self-attention mechanisms, employing standard and shifted window partitioning strategies, respectively. The shifted-window partitioning approach establishes links between neighboring non-overlapping windows in the previous layer and has exhibited effectiveness across various tasks like image classification, semantic segmentation, and object detection [55].

### 3.6 Fine-tuning the Hybrid Model

In developing our hybrid model, we employed advanced hyperparameter optimization techniques to ensure robust performance and high accuracy. Specifically, we utilized the Optuna framework to fine-tune the model's parameters through a systematic and rigorous process, optimizing key aspects to enhance the model's predictive capabilities. The optimization process began with the definition of an objective function aimed at maximizing the model's validation accuracy. This function systematically explored a comprehensive hyperparameter space, encompassing both transformer and convolutional layer parameters, to evaluate and identify the most effective configurations.

The hyperparameter space explored during optimization included various parameters critical to the model's architecture and training dynamics. These parameters ranged from transformer-specific settings, such as the inclusion of bias in query-key-value projections (qkv_bias), scaling factors (qk_scale), dropout rates (mlp_drop_rate, attn_drop_rate, proj_drop_rate, drop_path_rate), the number of attention heads (num_heads), embedding dimensions (embed_dim), and the size of the multi-layer perceptron (num_mlp), to convolutional layer configurations, including the number of input channels (in_channels_b0 to in_channels_b3), kernel sizes (c_m_b0 to c_m_b3), and dense layer parameters (dense_b0 to dense_b2, dropout_b0 to dropout_b2). The comprehensive range of these hyperparameters is detailed in Table **3**.

The optimization procedure was meticulously structured to ensure comprehensive exploration of the hyperparameter space while maintaining computational efficiency. We configured Optuna's TPE sampler with n_startup_trials=10 to facilitate initial exploration, followed by focused optimization based on the observed performance trends. A total of 20 trials were conducted, each representing a unique combination of hyperparameters evaluated for model performance. To ensure the reproducibility of the results, a fixed seed (seed=42) was set during the study initialization.

Each trial involved training the model for up to 100 epochs, incorporating early stopping based on validation performance to prevent overfitting. The Adam optimizer was employed with a learning rate of 0.001 and binary cross-entropy served as the loss function. Metrics monitored during training included validation accuracy and loss, providing a clear indicator of model performance.

Upon completion of the optimization trials, optuna successfully identified the best-performing hyperparameter configuration, as detailed in Table **4**, and the performance metrics of the optimized model are displayed in Table **5**. This optimal set of hyperparameters achieved a validation accuracy of 98.51%, demonstrating the effectiveness of the hyperparameter-tuning process in enhancing the model's predictive capabilities.

To provide a comprehensive overview of the optimization process, Table **6** presents a summary of validation accuracy and loss across all 20 trials conducted during the hyperparameter tuning process. This table underscores the performance outcomes of each trial, highlighting both successful and suboptimal configurations to offer insights into the optimization landscape.

The analysis of the hyperparameter impact reveals several key insights. The optimized dropout rates (mlp_drop_rate, attn_drop_rate, proj_drop_rate, and drop_path_rate) at 0.3262, 0.4107, 0.3871, and 0.3651 respectively, effectively mitigate overfitting while preserving the model's capacity to learn complex patterns. The selection of a smaller number of attention heads (num_heads = 4) and a reduced embedding dimension (embed_dim = 32) strikes an optimal balance between capturing sufficient contextual information and maintaining computational efficiency. Additionally, the convolutional layers configured with varying input channels and kernel sizes (primarily 3 and 5) enable the model to extract both fine-grained and broader features from the input data, enhancing its overall predictive performance.

Notably, trials 2, 14, 16, 17, and 20 achieved exceptionally high validation accuracies of 97.76%, 98.40%, 98.24%, 98.56%, and 98.56%, respectively. These configurations highlight the effectiveness of the selected hyperparameter ranges and the optimization strategy employed. Conversely, the majority of the other trials resulted in lower accuracies, underscoring the importance of precise hyperparameter tuning in achieving optimal model performance.

## 4 RESULTS

### 4.1 Experiment Setup

We carried out our experiments on the Kaggle platform, leveraging its computational resources. We used 73.1 GB of disk space to effectively store and manage datasets, model checkpoints, and the results of our experiments.

The Kaggle environment provides 13 GB of RAM, which facilitates efficient data loading and manipulation. We also utilized a GPU with 15.9 GB of memory to accelerate the training of deep-learning models. Additionally, Kaggle offers 19.5 GB of storage space for outputs, which helps in conveniently storing and analyzing model outputs, visualizations, and other data related to the experiments.

### 4.2 Performance Analysis

In this study, we compare the performance of our hybrid CNN-ViT model with the same model that uses only CNN layers. The comparison is conducted on the pneumonia dataset to evaluate the efficiency and effectiveness of both approaches in diagnosing pneumonia. The analysis of the performance of our models is presented in Table **7** and Fig. (**7**).

In Table **7**, The classification report provides a comprehensive evaluation of the model's performance across multiple metrics, precision, recall, f1-score, and support, for both the normal (class 0) and pneumonia (class 1) categories.

For the normal class, the model achieved a precision of 0.9738, which indicates a low false positive rate. The recall is a perfect 1.0000, indicating no false negatives. The f1-score is 0.9867, reflecting the model’s exceptional accuracy in classifying this category. The support for the Normal class is 297 instances.

In the pneumonia class, the model achieved a perfect precision of 1.0000, demonstrating no false positives and a recall of 0.9756, indicating a very low false negative rate. The f1-score for this class is 0.9877, showing strong performance. The support for the pneumonia class is 328 instances.

The model's overall accuracy is 0.9872. The macro average precision, recall, and f1-score are all approximately 0.987, indicating consistent performance across both classes. The weighted averages for these metrics are similarly aligned, underscoring the model's robustness.

The model excels in classifying both normal and pneumonia instances with high precision, recall, and f1-scores. The slight difference in recall between the classes suggests a minor area for improvement in identifying all pneumonia instances. However, these differences are minimal and do not significantly impact the model's overall performance.

In Fig. (**7**), the comparison between the CNN model and the hybrid model shows that the hybrid model consistently outperforms the CNN model across all performance metrics. Specifically, the hybrid model achieves an accuracy of 98.72% compared to the CNN model's 97.43%, a precision of 98.69% compared to 97.41%, a recall of 98.78% compared to 97.50%, and an F1 score of 98.72% compared to 97.44%. These results indicate that the hybrid model is more effective and reliable in classification tasks, offering better accuracy, precision, recall, and F1 score than the CNN model. When compared to several state-of-the-art models, our suggested method surpasses these models with a smaller number of parameters, as shown in Table **8**.

Fig. (**8**) provides a detailed view of the accuracy and loss metrics for both the hybrid model and the CNN model across the training and validation phases. The training and validation accuracy curves for the hybrid model show a rapid increase in the initial epochs, stabilizing around the 30th epoch with training accuracy nearing 98.78%. This suggests that the model quickly learns to classify the data correctly and maintains high performance without significant overfitting, as indicated by the close alignment of the training and validation accuracy curves. The training and validation loss curves also demonstrate a sharp decrease initially, leveling off around the same epoch as the accuracy curves. The low and stable loss values for both training and validation indicate that the model is not only accurate but also confident in its predictions.

For the CNN model, the training and validation accuracy curves exhibit a steady increase, achieving an accuracy of approximately 97.77%. The validation accuracy is slightly lower than the training accuracy, which could suggest that there is overfitting, but overall, the performance remains robust. The loss curves for the CNN model decrease consistently, with some fluctuations in the validation loss. The training loss stabilizes at a lower value than the validation loss, suggesting the model might be overfitting the training data.

Fig. (**9**) illustrates the confusion matrices for both the hybrid model and the CNN model, providing insight into their classification performance on the test dataset. The hybrid model shows a high number of correct classifications for both normal (295) and pneumonia (322) cases, with very few misclassifications (2 normal as pneumonia and 6 pneumonia as normal). This results in a high sensitivity and specificity, indicating that the hybrid model is highly accurate in distinguishing between normal and pneumonia cases. The CNN model also demonstrates good performance, with 293 correct normal classifications and 316 correct pneumonia classifications. There are slightly more misclassifications compared to the hybrid model (4 normal as pneumonia and 12 pneumonia as normal). The CNN model exhibits marginally lower sensitivity and specificity compared to the hybrid model.

### 4.3 Grad-CAM Visualization for Model Interpretability

To interpret the decision-making process of our models in detecting pneumonia, we employed Grad-CAM to generate visual heatmaps that reveal the region’s most influential in each model's classification. These visualizations provide valuable insights into how the models prioritize different areas of the input images.

**Fig. (10a-d):**
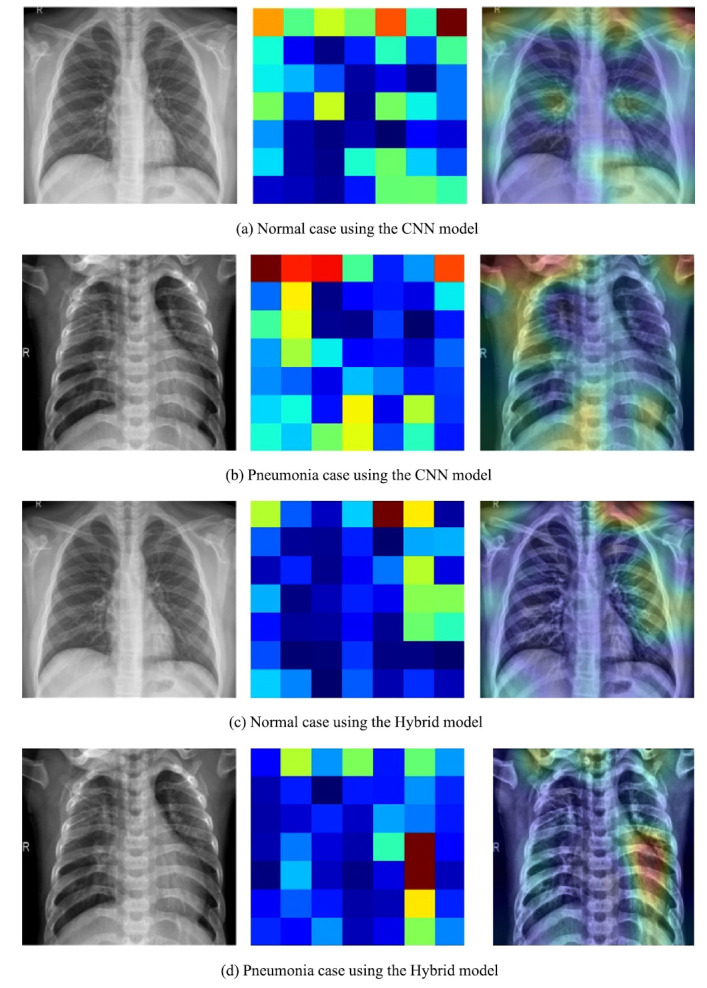
Grad-CAM visualization for the learning models.

Fig. (**10**) presents Grad-CAM heatmaps overlaid on chest X-ray images, comparing the CNN-only model and the hybrid CNN-VIT model in both normal and pneumonia cases. In the normal case using the CNN model (Fig. **10a**), the heatmap shows a broad focus, sometimes extending outside the lungs. This diffuse attention may indicate that the CNN model considers irrelevant areas, which could impact its performance. For the pneumonia case (Fig. **10b**), the CNN model again displays broad activation, capturing some lung regions associated with pneumonia but lacking precise focus, which may reduce accuracy.

In contrast, the hybrid CNN-VIT model demonstrates a more localized focus on medically relevant areas. For the normal case (Fig. **10c**), the model primarily centers on the lungs, effectively ignoring extraneous regions, which suggests a more reliable classification process. In the pneumonia case (Fig. **10d**), the hybrid model highlights regions within the lungs associated with signs of pneumonia, such as opacities and consolidations. This precise focus reflects the model's ability to detect subtle pathological signs accurately.

The Grad-CAM visualizations reveal that the hybrid CNN-VIT model achieves higher accuracy and interpretability by focusing on clinically relevant regions, while the CNN-only model's broader activation may contribute to slightly lower performance. By concentrating on medically significant features, the hybrid model enhances prediction reliability and validation, ensuring that classifications are based on meaningful patterns rather than irrelevant correlations.

## 5 DISCUSSION

This study introduces a novel approach for pneumonia detection using a hybrid architecture that combines CNNs with VITs. This combination leverages the strengths of both models, effectively enhancing feature extraction and improving classification accuracy. The methodology encompasses several innovative steps, including data preprocessing with CLAHE, data augmentation, and hyperparameter optimization using Optuna.

The application of CLAHE to enhance image features is a critical step in this process. This technique improves the visibility of finer details within the chest x-ray images, thus aiding in more accurate feature extraction by the CNNs. Additionally, data augmentation techniques such as horizontal flipping, rotation, and zooming ensure the robustness of the model by providing diverse training examples. This step is essential in mitigating overfitting, particularly given the imbalance in the dataset.

The hybrid model architecture integrates a CNN backbone with swin transformer blocks. The CNN layers perform initial feature extraction, capturing local patterns within the images. These extracted features are then processed by the swin transformer blocks, which are adept at handling long-range dependencies and capturing global context through self-attention mechanisms. This hierarchical processing, involving patch extraction and embedding followed by window-based and shifted window self-attention, allows the model to understand complex visual patterns more effectively.

optuna’s bayesian optimization, using the TPE sampler, plays a crucial role in fine-tuning the model. This systematic approach explores various configurations of hyperparameters, optimizing dropout rates, the number of attention heads, embedding dimensions, and other critical parameters. The rigorous optimization process ensures that the model achieves its best possible performance.

The experimental setup on the Kaggle platform, leveraging its computational resources, yielded remarkable results. The proposed hybrid CNN-VIT model achieved an accuracy of 98.72%, significantly outperforming the baseline CNN model. Detailed analysis shows that the hybrid model consistently excels in key performance metrics, including precision, recall, and F1-score. This indicates not only the model’s high accuracy but also its reliability in correctly classifying pneumonia cases.

To demonstrate the effectiveness and necessity of each module in the proposed hybrid CNN-VIT architecture, ablation experiments were conducted by integrating VIT with several state-of-the-art pretrained models. The performance of the hybrid model was systematically compared against these individual pretrained models, both with and without the addition of VIT. As presented in Table **9**, the hybrid CNN-VIT model consistently outperforms the standalone pretrained models, achieving higher accuracy and other evaluation metrics. For example, while DenseNet201, VGG16, and GoogleNet exhibit strong performance on their own, their VIT-enhanced counterparts show marked improvements, yet the hybrid CNN-VIT model still surpasses all, underscoring the superior capability of the combined architecture in handling the pneumonia classification task.

Furthermore, the confusion matrices for both the hybrid CNN-VIT model and the standard CNN model provide deeper insights into their classification performance. The hybrid model demonstrates enhanced sensitivity and specificity, with significantly fewer misclassifications compared to the individual models. This reinforces the robustness and reliability of the hybrid approach. Additionally, the stability of accuracy and loss metrics across both training and validation phases for the hybrid model indicates efficient learning processes and minimal overfitting, highlighting the advantage of incorporating VIT into the pretrained frameworks.

### 5.2 Application and Profitable Implications

The aim of this proposed research is to develop a refined deep-learning model specifically for the accurate classification of pneumonia, leveraging the capabilities of advanced deep-learning technology. The potential applications of this research are extensive and include:

#### 5.2.1 Improved Pneumonia Diagnosis

This research provides significant support to radiologists and clinicians in accurately diagnosing pneumonia using medical imaging data such as chest X-rays. Ensuring consistent and exact classification findings reduces the chance of misinterpretation and improves early disease detection.

#### 5.2.2 Tailored Treatment Planning

Precise pneumonia classification supports the development of personalized treatment plans tailored to individual patients, enhancing the effectiveness of medical care.

#### 5.2.3 Clinical Decision Support System

The model developed in this research can serve as a clinical decision support tool, assisting healthcare providers in making well-informed patient management decisions. It offers reliable and accurate pneumonia classification results, thereby supporting precise and personalized patient care strategies.

This research has significant potential to enhance pneumonia diagnosis, personalize treatment planning, support research initiatives, and serve as a valuable clinical decision-making tool. These applications can positively impact patient care, improve clinical outcomes, and contribute to advancements in medical fields like neuro-oncology.

Furthermore, this innovative research is poised to transform the medical imaging domain, delivering considerable societal benefits. The proposed deep learning model, through accurate pneumonia classification, employs advanced reconstruction and fine-tuning techniques. Its direct clinical applicability and seamless integration into existing workflows facilitate its implementation in clinical environments, thereby improving pneumonia diagnosis, treatment planning, and patient outcomes.

Additionally, the approach minimizes the need for extensive manual annotation, reducing both costs and development time, making it highly suitable for clinical use. Beyond its clinical implications, this research holds significant societal value by enhancing patient care, reducing healthcare costs, and addressing global healthcare challenges.

A potential future direction can be the application of a modified version of the Hybrid CNN-VIT model for the segmentation of the liver and kidneys from various image modalities. This is because, although there are different deterministic and probabilistic approaches, an effective automated method is still needed to achieve their accurate segmentations by utilizing VITs. Exploring this application could extend the model's utility beyond pneumonia classification, addressing critical needs in the segmentation of vital organs across diverse imaging techniques. Moreover, there is an intention to enhance the proposed DL model by incorporating more advanced hybrid ensemble techniques with newly available chest x-ray datasets. Additionally, the plan is to implement explainable AI techniques to offer deeper insights into the decision-making process of the DL model. This, in turn, aims to enhance the confidence and trust of clinicians and patients in the diagnostic process.

Despite the model's enhanced precision, there are limitations that should be acknowledged. The need for clearer images and a more advanced deep learning architecture limits the ability to achieve even higher performance. Additionally, the hybrid model, while efficient, may require further improvements in image processing techniques to enhance its effectiveness for this task. These limitations highlight areas for future improvement, especially as advancements in deep learning and medical imaging become available.

## CONCLUSION

This article introduces a new method in DL for classifying pneumonia diseases. The integration of CNNs with VIT allows for effective capture of both local and global features, leading to superior classification performance. Key innovations include the application of CLAHE for enhanced image preprocessing, advanced data augmentation techniques to increase model robustness, and meticulous hyperparameter optimization using Optuna.

The hybrid model architecture is a standout feature of this study. The CNN backbone performs the initial feature extraction, capturing fine-grained local patterns from the chest x-ray images. These features are then segmented into patches and processed by swin transformer blocks, which employ window-based and shifted window self-attention mechanisms to capture long-range dependencies and global context within the images. This hierarchical processing approach enables the model to understand complex visual patterns more effectively. CLAHE is applied to the images to enhance their contrast and make finer details more visible. This preprocessing step is crucial for improving the quality of the input data, thereby aiding the feature extraction process performed by the CNN layers. The data augmentation techniques, including horizontal flipping, rotation, and zooming, ensure the model is exposed to a diverse set of training examples, mitigating overfitting and enhancing generalization.

Hyperparameter optimization is another critical component of this study. Using optuna’s bayesian optimization framework, the model’s parameters are fine-tuned systematically. This includes optimizing dropout rates, the number of attention heads, embedding dimensions, and other essential parameters. The TPE sampler efficiently explores the hyperparameter space, ensuring the model achieves optimal performance.

By employing a chest x-ray dataset, we demonstrated that our proposed model is highly effective in diagnosing pneumonia with high accuracy. The model achieved a pneumonia classification accuracy of 98.72%, with a loss of 0.064. Further analysis showed that our model surpasses other models and existing methods, especially in terms of precision. We foresee that our developed model can be implemented in clinical settings to speed up and improve the accuracy of pneumonia diagnosis. In light of the conclusion, the paper has achieved the following milestones:

### Innovative Model Architecture

Successfully developed a hybrid CNN-VIT model that leverages the strengths of both convolutional networks and vision transformers for superior pneumonia detection.

### Enhanced Preprocessing Techniques

Implemented CLAHE to improve the quality of chest x-ray images, aiding in more accurate feature extraction.

### Robust Data Augmentation

Applied advanced data augmentation techniques to mitigate overfitting and enhance the model’s generalization capabilities.

### Optimal Hyperparameter Tuning

Utilized optuna for meticulous hyperparameter optimization, ensuring the model operates at its best performance.

### Cross-validation

Conducted a 5-fold cross-validation method, achieving an average accuracy of 98.07%, demonstrating the model's consistency and robustness.

### High Accuracy and Robustness

Achieved an impressive accuracy of 98.72%, demonstrating the model's high reliability and robustness in detecting pneumonia from chest X-ray images.

### Resource-efficient Deployment

Designed a lightweight model suitable for deployment in resource-constrained settings, ensuring broader applicability and accessibility.

### Superior Performance

Outperformed existing state-of-the-art models, highlighting the effectiveness and innovation of the proposed approach.

### Comprehensive Evaluation

Provided detailed insights through confusion matrices, stability of accuracy, and loss metrics, affirming the model’s efficiency and reliability.

## Figures and Tables

**Fig. (1):**
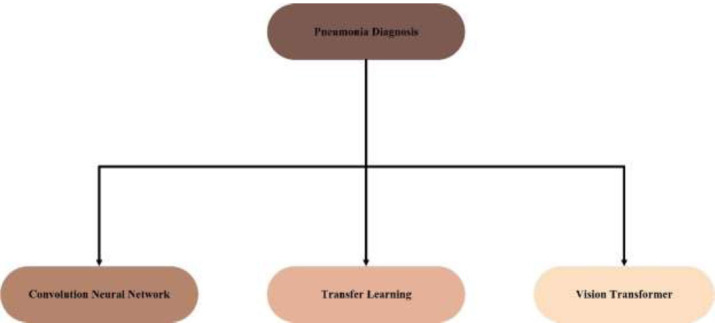
The tree diagram of related works for pneumonia diagnosis.

**Fig. (2):**
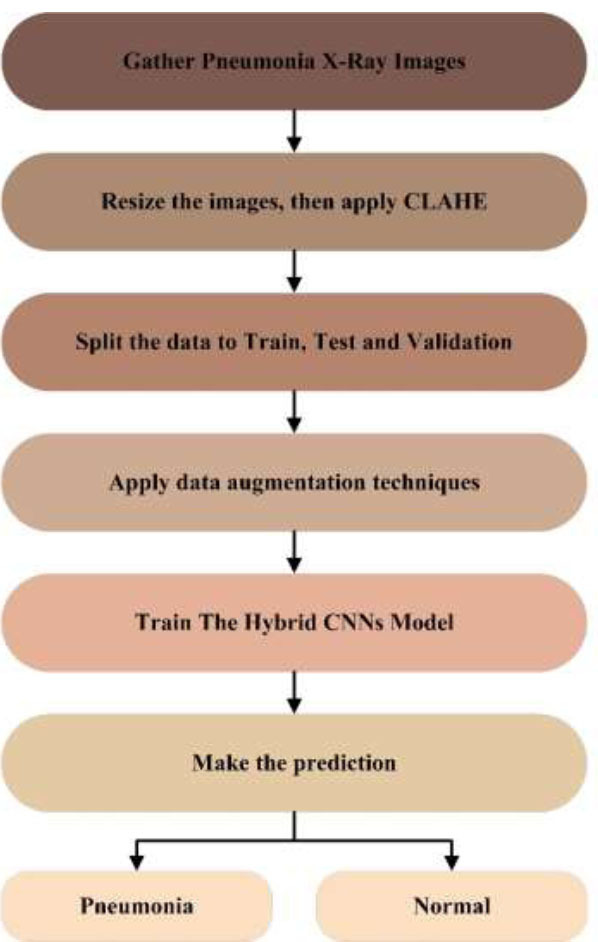
Diagram of our proposed research.

**Fig. (3):**
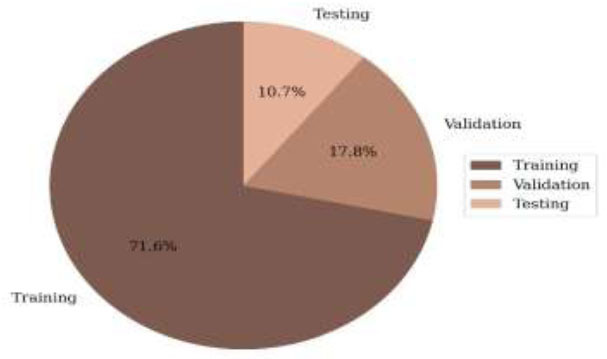
The distribution of the pneumonia dataset.

**Fig. (4a, b):**
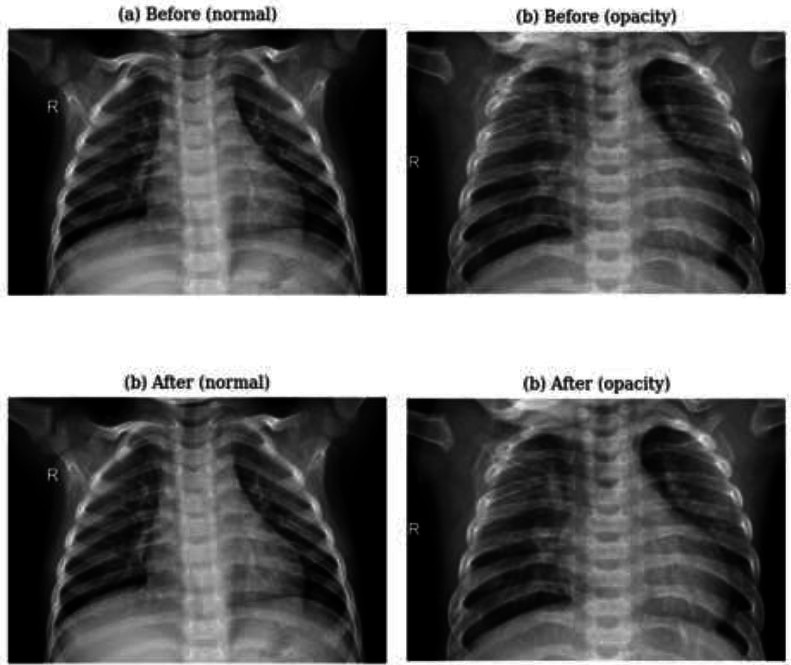
Chest x-ray images of normal cases and pneumonia cases before and after image preprocessing.

**Fig. (5):**
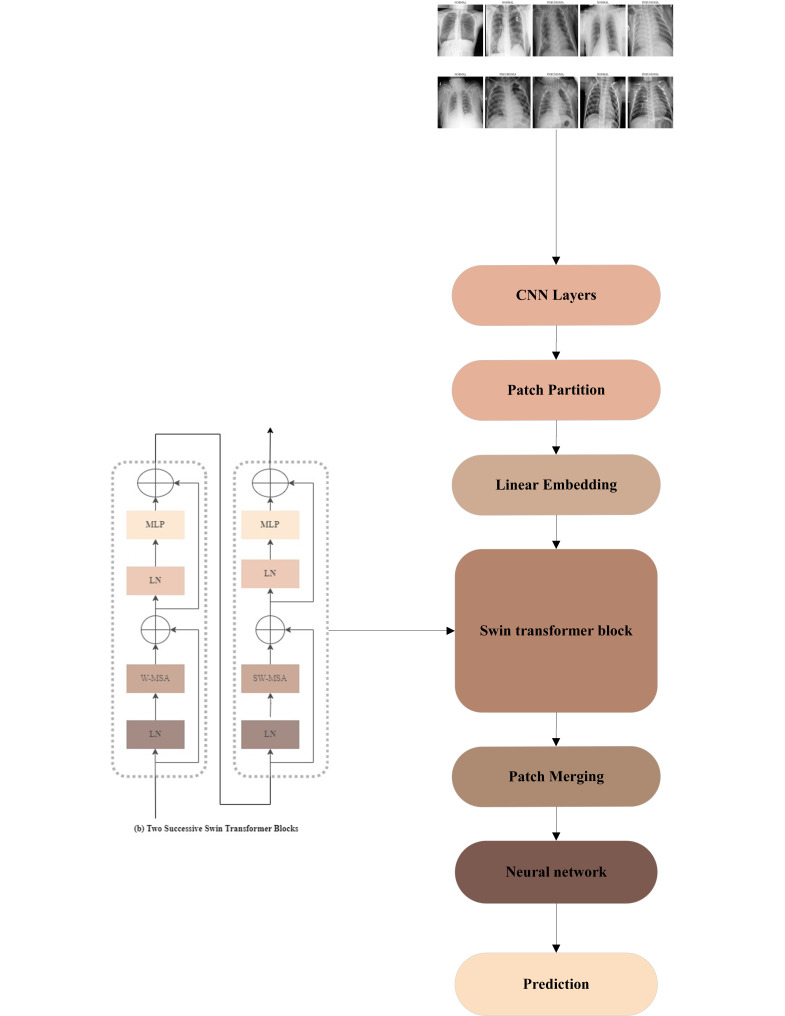
Proposed architecture of the learning model.

**Fig. (6):**
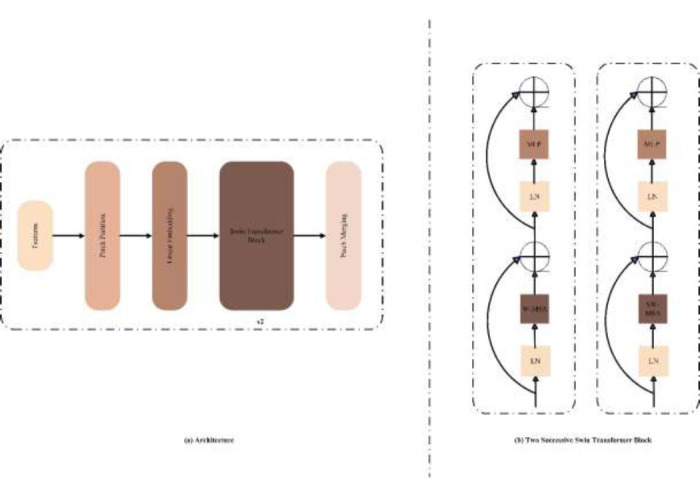
(**A**) The architecture of the implemented one stage swin transformer, (**B**) two successive swin transformer blocks.

**Fig. (7):**
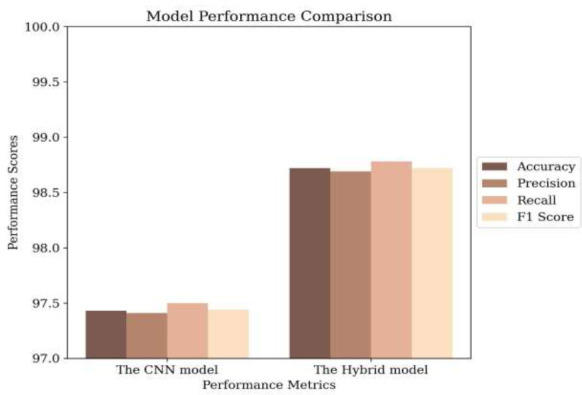
Analysis of the learning model.

**Fig. (8):**
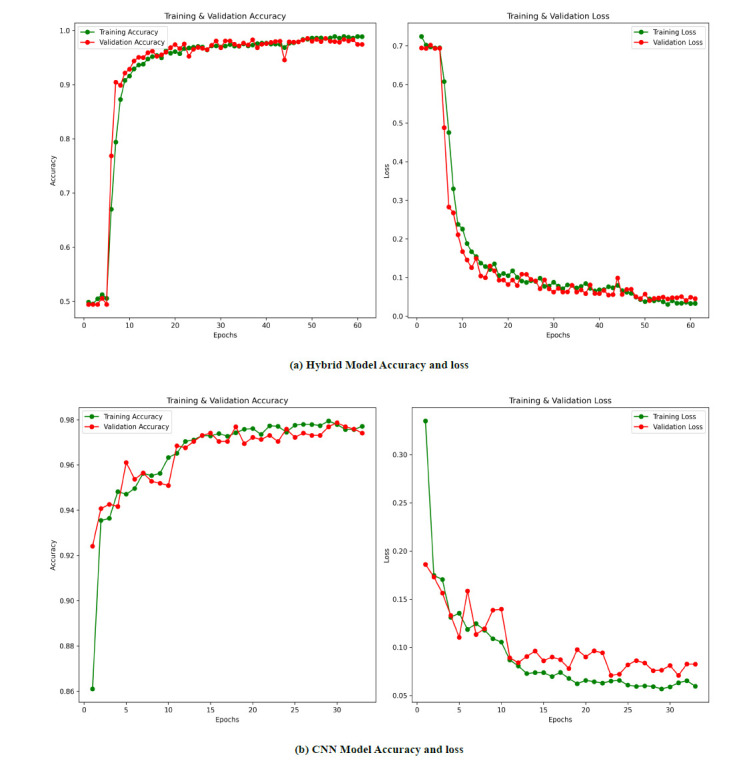
Accuracy and loss for the learning models.

**Fig. (9a,b):**
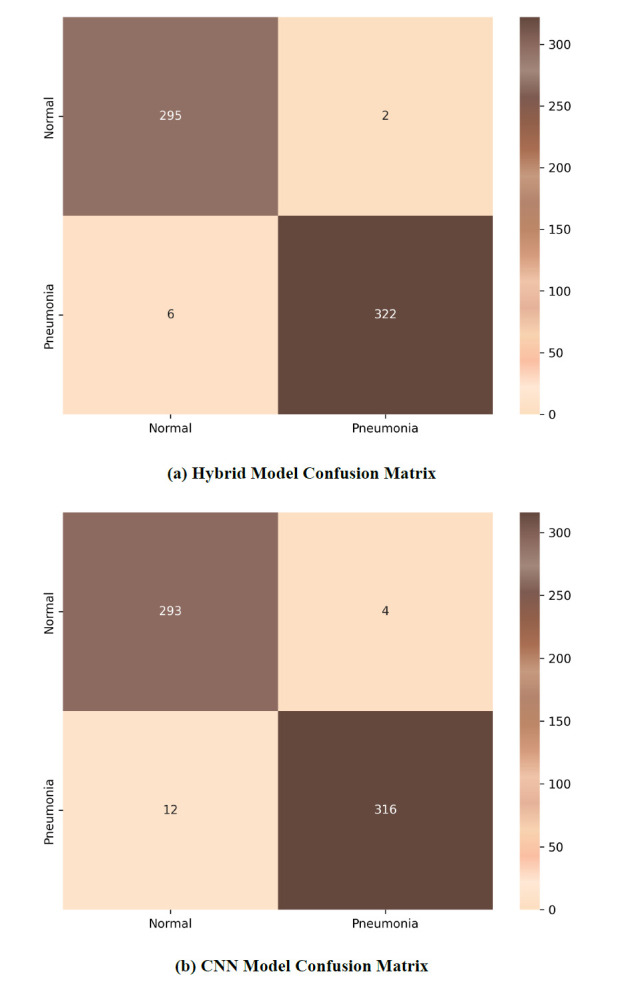
Confusion matrix for the learning models.

**Table 1:** Comparative analysis of deep learning techniques for pneumonia diagnosis.

Study	Method/Network	Result	Disadvantage/Limitation
Stephen *et al*. [25]	CNN (Custom)	Training accuracy: 95.31%, Validation accuracy: 93.73%	Generalizability limited by restricted dataset
Yue *et al*. [26]	CNN (CT Scan features)	AUC: 97%	Limited dataset
Liang *et al*. [27]	CNN with residual blocks and dilated convolution layers	Recall: 96.7%, F1-score: 92.7%	-
Yadav *et al*. [28]	CNN	Accuracy: 84.18%, Recall: 78.33%, F1-score: 85.66%	-
Harsh *et al*. [29]	CNN with various filter sizes (Ensemble)	Recall: 99.23%	Overfitting and inaccuracies
Ebru *et al*. [30]	Deep learning framework	Accuracy: 88.62%, Recall: 97.43%	-
Urey *et al*. [31]	CNN (feature extraction)	Accuracy: 79%	Limited accuracy, low classification performance
Zhang *et al*. [32]	CNN with confidenceaware module	AUC: 83.61%	Limited in generalizing anomaly detection
Chouhan *et al*. [33]	Transfer Learning	Accuracy: 96.4%	-
Rajpurkar *et al*. [34]	DenseNet-121 (Transfer Learning)	F1 score: 76.8%	-
Jain *et al*. [35]	Transfer Learning + CNN	Accuracy: 95.62%, Recall: 95%, Precision: 96%	-
Asnaoui *et al*. [36]	E3CC and VGG16 + CapsNet	E3CC: Accuracy 81.54%, VGG16 + CapsNet: 88.30%	-
Mittal *et al*. [37]	Simple CapsNet (Transfer Learning)	Accuracy: 85.26%, Recall: 94%, F1-score: 89%	-
Ayan *et al*. [38]	VGG16, Xception (Transfer Learning)	Accuracy: 87%	-
Kundu *et al*. [39]	Ensemble of GoogLeNet, ResNet-18, DenseNet-121	Accuracy: 86.85%	-
O'Quinn *et al*. [40]	AlexNet (Transfer Learning)	Accuracy: 76%	-
Talo [41]	ResNet152 (Transfer Learning)	Accuracy: 97.4%	No preprocessing or feature extraction required
Varshni *et al*. [42]	DenseNet + SVM	AUC: 80.02%	Generalizability concerns due to dataset limitations
Sukhendra *et al*. [50]	VIT	Accuracy: 97.61%	-
Usman *et al* [51]	VIT	Accuracy: 87%	-

**Table 2:** Augmentations used in our study and their corresponding values.

Methods	Corresponding Parameters
Zoom range	0.2
Rotation range	15
Horizontal flip	True
Rescaling	1.0/255

**Table 3:** Hyperparameter space explored using optuna.

Hyperparameter	Description	Range / Options
qkv_bias	Include bias in query-key-value projections	True, False
qk_scale	Scaling factor for query-key normalization	0.1 to 2.0 (continuous)
mlp_drop_rate	Dropout rate for the multi-layer perceptron	0.0 to 0.8 (continuous)
attn_drop_rate	Dropout rate for the attention mechanism	0.0 to 0.8 (continuous)
proj_drop_rate	Dropout rate for the projection layers	0.0 to 0.8 (continuous)
drop_path_rate	Dropout rate for the path	0.0 to 0.8 (continuous)
num_heads	Number of attention heads	4 to 32 (step of 4)
embed_dim	Embedding dimension	num_heads * 8 to num_heads * 128 (step of num_heads * 8)
num_mlp	Number of nodes in the MLP layer	embed_dim / 4 to embed_dim / 2 (step of embed_dim / 16)
in_channels_be	Number of input channels for convolutional block 0	16 to 128 (step of 16)
in_channels_b1	Number of input channels for convolutional block 1	128 to 256 (step of 16)
in_channels_b2	Number of input channels for convolutional block 2	256 to 384 (step of 16)
in_channels_b3	Number of input channels for convolutional block 3	384 to 512 (step of 16)
c_m_be	Kernel size for convolutional layer 0	3 to 9 (step of 2)
c_m_b1	Kernel size for convolutional layer 1	3 to 9 (step of 2)
c_m_b2	Kernel size for convolutional layer 2	3 to 9 (step of 2)
c_m_b3	Kernel size for convolutional layer 3	3 to 9 (step of 2)
dense_b0	Number of units in dense layer 0	256 to 512 (step of 64)
dense_b1	Number of units in dense layer 1	128 to 256 (step of 32)
dense_b2	Number of units in dense layer 2	16 to 128 (step of 16)
dropout_be	Dropout rate in dense layer 0	0.1 to 0.9 (step of 0.1)
dropout_b1	Dropout rate in dense layer 1	0.1 to 0.9 (step of 0.1)
dropout_b2	Dropout rate in dense layer	0.1 to 0.9 (step of 0.1)

**Table 4:** Best hyperparameter configuration identified by optuna.

Hyperparameter	Best Value
qkv_bias	False
qk_scale	1.0299
mlp_drop_rate	0.3262
attn_drop_rate	0.4107
proj_drop_rate	0.3871
drop_path_rate	0.3651
num_heads	4
embed_dim	32
num_mlp	8
in_channels_b0	96
in_channels_b1	192
in_channels_b2	336
in_channels_b3	480
c_m_b0	5
c_m_b1	3
c_m_b2	3
c_m_b3	5
dense_b0	384
dense_b1	160
dense_b2	64
dropout_b0	0.4
dropout_b1	0.4
dropout_b2	0.1

**Table 5:** Performance metrics of the optimized model.

Metric	Value
Validation accuracy	98.56%
Test accuracy	98.72%
Training loss	0.042
Validation loss	0.038

**Table 6:** Summary of validation accuracy and loss across 20 optimization trials.

Trial	Validation Accuracy (%)	Validation Loss
1	47.52	0.6932
2	97.76	0.06499
3	47.52	0.6932
4	47.52	0.6932
5	47.52	0.6932
6	52.48	0.6932
7	47.52	0.6932
8	47.52	0.6932
9	52.48	0.6931
10	52.48	0.6931
11	47.52	0.6931
12	47.52	0.6931
13	47.52	0.6932
14	98.40	0.0554
15	52.48	0.6931
16	98.24	0.04886
17	98.56	0.05911
18	52.48	0.6931
19	47.52	0.6932
20	98.56	0.05292

**Table 7:** Classification report of the proposed model.

-	Precision	Recall	F1-score	Support
Normal (Class 0)	0.9738	1.0000	0.9867	297
Pneumonia (Class 1)	1.0000	0.9756	0.9877	328
accuracy	-	-	0.9872	625
macro avg	0.9869	0.9878	0.9872	625
weighted avg	0.9875	0.9872	0.9872	625

**Table 8:** The comparison analysis of pneumonia classification.

Refs.	Year	AI Model Architecture	ACC (%)
Ayan *et al*. [38]	2019	VGG16 Xception	87 82
Chouhan *et al*. [33]	2020	CNN	96
Yadav *et al*. [28]	2019	CapsNet	83
Liang *et al*. [27]	2020	CNN VGG16	91 74
Asnaoui *et al*. [36]	2021	CNN	84
Mittal *et al*. [37]	2020	E3CC VGG16 + CapsNet	82 88
Jain *et al*. [35]	2020	CNN	85
ERDEM *et al*. [30]	2020	CNN	87
Darici *et al*. [56]	2020	CNN Ensemble	95 95
Talo *et al*. [41]	2019	ResNet 152	97
O'Quinn *et al*. [40]	2019	AlexNet	76
Urey *et al*. [31]	2019	ResNet	78
Stephen *et al*. [25]	2019	CNN	93
Khalid *et al*. [36]	2020	CNN VGG16 VGG19 InceptionV3 Xception DenseNet201 MobileNetV2 InceptionResNetV2 ResNet50	84 86 86 95 83 94 96 96 97
Mohammad *et al*. [57]	2021	ResNet50 Compound Scaled ResNet50	97 98
Juan *et al*. [58]	2020	Xception CNN	97.3
Usman *et al* [51]	2022	VIT	87
Sukhendra *et al*. [50]	2024	VIT	97.61
The proposed model (Hybrid CNN-VIT)	2024	Hybrid CNN-VIT	98.72

**Table 9:** Classification evaluation performance of the individual pretrained models.

Pretrained Models	ACC	SEN	SPE	PRE	F1-score	AUC
VGG16	95.85%	93.78%	96.89%	93.92%	93.78%	95.33%
VGG16 + VIT	96.50%	94.53%	97.20%	94.51%	94.58%	96.05%
Xception	95.79%	93.69%	96.84%	93.97%	93.74%	95.27%
Xception + VIT	96.48%	94.37%	97.13%	97.16%	94.43%	95.96%
EfficientNetB7	92.76%	89.13%	94.57%	92.14%	89.30%	91.85%
EfficientNetB7 + VIT	93.55%	91.02%	95.36%	93.03	90.01%	92.74%
GoogleNet	94.46%	91.69%	95.84%	92.14%	91.78%	93.77%
GoogleNet + VIT	95.15%	92.58%	96.53%	93.03%	92.59%	94.46%
InceptResNetV2	94.44%	91.67%	95.83%	91.97%	91.69%	93.75%
InceptResNetV2 + VIT	95.35%	92.56%	96.34%	92.88%	92.58%	94.34%
DenseNet201	96.95%	95.42%	97.71%	95.51%	95.44%	96.57%
DenseNet201 + VIT	97.51%	96.03%	98.12%	96.10%	96.35%	97.26%
AlexNet	95.49%	94.97%	96.15%	95.13%	95.97%	96.10%
AlexNet + VIT	96.58%	95.88%	96.28%	95.97%	96.12%	96.62%
The CNN mode	97.43%	97.50%	97.87%	97.41%,	97.44%	97.58%
The proposed model (Hybrid CNN-VIT)	98.72	98.17	99.33	98.69	98.72	99.82

## Data Availability

All data generated or analyzed during this study are included in this published article.

## LIST OF ABBREVIATIONS

CNNs: Convolutional Neural Networks
CT: Computed Tomography
NLM: Non-Local Means
DL: Deep Learning
TL: Transfer Learning
ViTs: Vision Transformers
GELU: Gaussian Error Linear Units
LN: Layer Norm
